# Deciphering the role of SMU.1147 in peptide-mediated signaling and competence in *Streptococcus mutans*

**DOI:** 10.1128/spectrum.02917-24

**Published:** 2025-03-05

**Authors:** Jeong Nam Kim, Si-Uk Ryoo, Yeuna Nam

**Affiliations:** 1 Department of Integrated Biological Science, Pusan National University, Busan, South Korea; 2 Department of Microbiology, College of Natural Sciences, Pusan National University, Busan, South Korea; University of Florida College of Dentistry, Gainesville, Florida, USA

**Keywords:** *Streptococcus mutans*, competence stimulating peptide (CSP), transcriptome analysis, peptide-mediated signaling, SMU.1147

## Abstract

*Streptococcus mutans* is a primary cariogenic pathogen involved in dental biofilm formation, a major virulence factor in the development of dental caries. In *S. mutans*, the competence-stimulating peptide (CSP), encoded by *comC*, plays a critical role in environmental stress response, growth regulation, and virulence expression. In this study, we performed transcriptome analysis to investigate the role of SMU.1147, a unique core gene in *S. mutans*, in biological pathways related to transport, defense responses, and environmental sensing. The deletion of SMU.1147 led to the upregulation of genes involved in carbohydrate uptake and metabolism, particularly phosphotransferase system (PTS) transporters, thereby enhancing the sugar transport capacity. However, despite increased sugar uptake, the mutant strain did not show significant changes in growth rate or ATP production and displayed slightly reduced organic acid production. Additionally, the mutant exhibited significantly reduced cell viability after an 8-h incubation compared to the parental strain. Notably, genes associated with CSP-dependent signal transduction and stress defense, such as *comX*, *comR*, *htrA*, *scnRK*, and *ciaRH*, were downregulated in the mutant strain. Furthermore, stress-related genes, including *spxA2*, *clpP*, and *clpX*, were significantly downregulated, suggesting compromised protein quality control and oxidative stress responses. Our findings suggest that SMU.1147 plays a critical role in regulating peptide-mediated signaling, metabolic coordination, and environmental adaptation in *S. mutans*, positioning it as a key integrator of the metabolic and stress response networks that are essential for pathogenicity and survival.

**IMPORTANCE:**

Understanding the regulatory mechanisms that govern virulence and environmental adaptation in *Streptococcus mutans* is essential for developing strategies to mitigate dental caries. This study reveals the critical role of the SMU.1147 gene in *S. mutans* in metabolic regulation, stress response, and cell viability. Our results demonstrate how the deletion of this gene affects sugar uptake and organic acid production, leading to imbalances in carbon metabolism and reduced long-term survival. These findings provide valuable insights into the ability of *S. mutans* to adapt to stressed conditions and highlight the role of SMU.1147 in modulating biofilm formation and virulence, contributing to our understanding of regulatory pathways in dental pathogens.

## INTRODUCTION

Understanding the development of dental caries continues to evolve as new knowledge emerges from basic and clinical research (1). It is widely acknowledged that biofilms on the hard tissues of the mouth undergo cycles of acidification, leading to tooth demineralization. These acidification periods are followed by alkalinization phases, in which the pH returns to near neutrality. During the alkalinization of plaque and the fasting period, remineralization of tooth enamel occurs via normal repair processes affected by saliva (2). Caries initiation or progression occurs when demineralization outweighs remineralization phases. In addition, the composition of the microbial flora on healthy surfaces of the teeth differs in meaningful ways from those populations found in carious lesions. Specifically, cariogenic microflora are characterized by increased proportions of highly acidogenic and aciduric species, such as *S. mutans*, lactobacilli, and other acid-tolerant bacteria (3–8), with *S. mutans* consistently showing the strongest association with human dental caries. To persist and emerge in significant numbers, cariogenic bacteria must be able to compete effectively for limiting sources of carbon and energy and adapt rapidly to changes in nutrient sources and availability. Potent bi-directional antagonistic interactions between *S. mutans* and commensals also affect the progression (9, 10).

A critical process in the formation of biofilms across a wide range of bacteria involves the capacity to engage in intercellular communication, which is affected by the sensing of secreted small molecules. This process, known as quorum sensing, controls a wide variety of essential functions that contribute to the establishment and persistence of single-species and complex biofilms (11–13). Quorum sensing also regulates many physiological, biochemical, and surface properties of bacteria, often in ways essential for pathogenesis. Peptide-based quorum sensing is of major importance for the predominant human dental caries pathogen, *S. mutans*. Substantial efforts have focused on what was initially discovered as a quorum-sensing pathway for the control of genetic competence. This system includes a small peptide encoded by *comC* that is externalized by a dedicated secretion pathway (ComAB) and sensed by the ComDE two-component signal transduction system (TCS) (14). The addition of as little as 30 nM competence-stimulating peptide (CSP) to cultures of *S. mutans* growing in complex medium can enhance transformation efficiency by 100-fold or more. Recently, substantial progress has been made in the ComRS signaling pathway, which is composed of an Rgg-type transcriptional activator encoded by *comR* and a small hydrophobic peptide encoded by *comS*. The 18-amino acid (aa) ComS peptide is externalized and processed to yield the 7-aa *comX* or *sigX* inducing peptide, XIP; ComX (SigX) being an alternative sigma factor that is necessary for the activation of late competence genes encoding the proteins for DNA uptake and metabolism (15, 16). Unlike many peptide-based quorum-sensing systems that function through membrane-bound signal transduction complexes, XIP is re-internalized through a general oligopeptide permease and binds to ComR to activate *comX* transcription (16). The importance of CSP and XIP in the regulation of gene expression and virulence cannot be overstated since they are central regulators of a variety of phenotypes that are essential for the ability of *S. mutans* to establish and persist in oral biofilms; to tolerate acid and other stressors; to form biofilms; to produce bacteriocins; and to regulate DNA uptake, extracellular DNA release, and programmed cell death (14, 17–22).

As part of the analysis of the gene content of *S. mutans*, important core genes called the “unique core genome (UCG)” of *S. mutans* have been identified (23). These UCGs influence various aspects of *S. mutans*, such as growth, stress tolerance, biofilm formation, and competence (23–25). Among these, the SMU.1147c gene, encoding a 61-aa peptide (called ScnC), is found in all strains of *S. mutans* examined thus far. It also appears to be unique to this bacterium, as a similar protein has not been found in many organisms available genome sequences (26). This gene is transcribed along with TCS, *scnK* (histidine kinase) and *scnR* (response regulator) in the operon structure (24). The *scnRK* genes, carrying GenBank designations SMU.1146 and SMU.1145, encode a TCS that has not yet received much attention. One report indicated that ScnRK contributes to the ability of the bacteria to cope with H_2_O_2_ stress and resists killing by macrophages (27). ScnK, also known as HK3 (28), has a modest effect on acid tolerance. In addition, an apparent ortholog of this system in *Streptococcus pyogenes* appears to influence bacteriocin expression (29). A previous study has indicated that ScnKR enhances resistance to acid, hydrogen peroxide stress, and macrophages (27), whereas SMU.1147 influences virulence-related phenotypes through peptide-based intercellular communication pathways (24). The disruption of SMU.1147 results in reduced biofilm formation, slower growth under acidic or oxidative stress conditions, and a significant decrease in genetic competence, including lower expression of *comX* and *comY* (24). Intriguingly, competence defects could not be rescued by external competence signals such as XIP or CSP.

The aim of this study was to elucidate the mechanisms by which SMU.1147 modulates bacterial behavior with the goal of leveraging this knowledge to develop more effective strategies for controlling the cariogenic potential of *S. mutans*. Our findings indicated that 349 genes and operons involved in virulence and persistence were significantly altered in the SMU.1147 mutant strain compared to those in the parental strain. Additionally, efforts have been made to establish a connection between gene expression and competence, further highlighting the biological role of SMU.1147 as a signaling molecule.

## RESULTS

### Contribution of SMU.1147 to gene expression in *S. mutans*

Previous studies have demonstrated that the SMU.1147 deletion mutant strain of *S. mutans* exhibits reduced tolerance to various stressors, decreased biofilm formation, and impaired genetic competence (24). The product of the SMU.1147 gene, identified as a peptide, is in both the cell wall and cytoplasmic fractions, suggesting involvement in novel peptide-mediated signaling pathways and CSP- or XIP-dependent competence systems. In this study, RNA-seq analysis was performed to compare the gene expression profiles of the SMU.1147 mutant and wild-type (WT) *S. mutans* strains with the aim of elucidating the relationship between gene expression and observed phenotypic changes.

**Fig 1:**
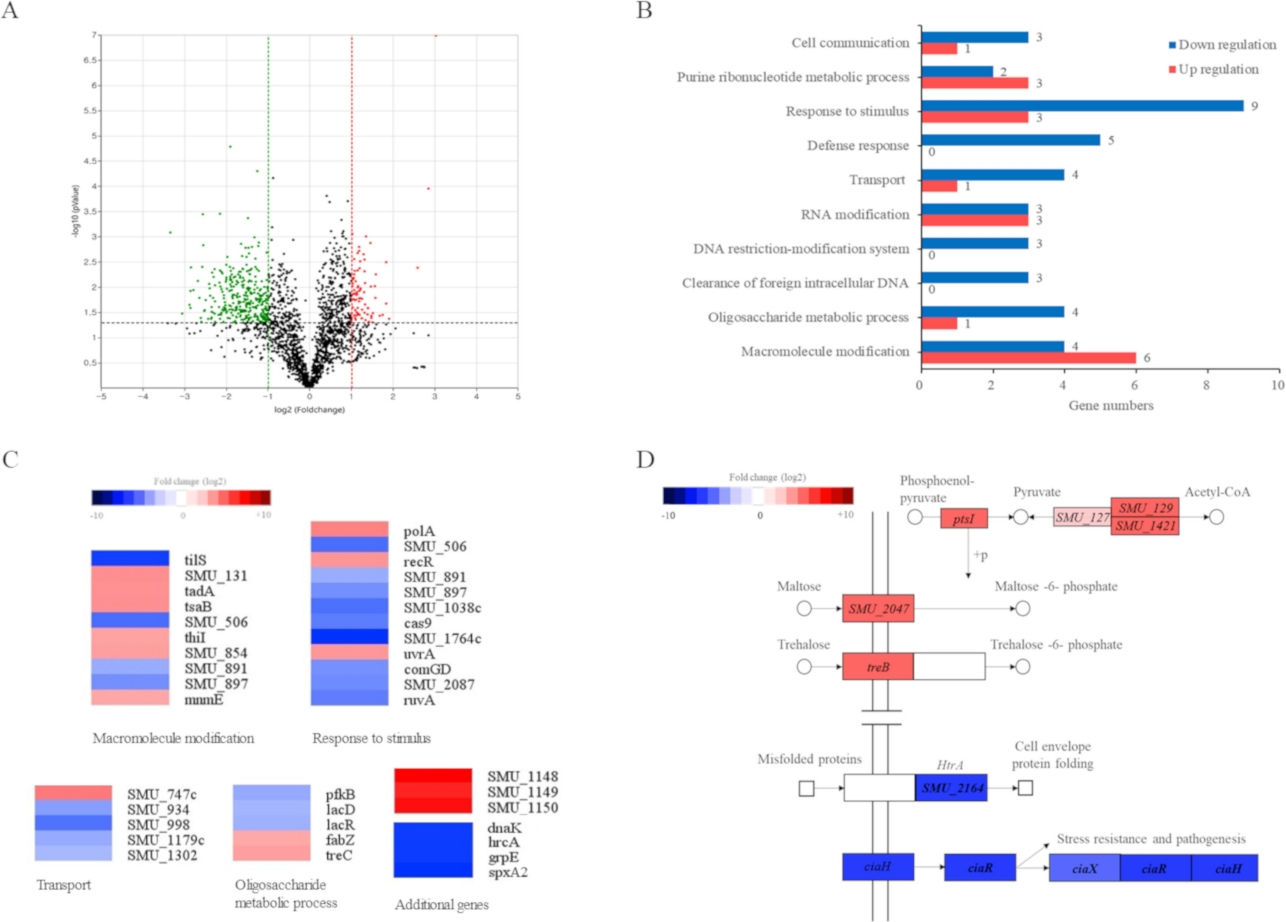
Differential gene expression in ΔSMU.1147 mutant identified by RNA-seq. (**A**) Volcano plot illustrating differential gene expression. The x-axis represents log2 fold change, and the y-axis denotes log10 *P*-value. Red and green dots indicate genes upregulated and downregulated by more than twofold, respectively (*P* < 0.05). (**B**) Pathway analysis was conducted using the Database for Annotation, Visualization, and Integrated Discovery (DAVID) bioinformatics tool based on gene expression data. Genes were categorized into biological pathways (Expression Analysis Systematic Explorer [EASE] score < 0.05). Blue and red bars represent downregulation and upregulation, respectively. (**C**) Heatmap analysis performed using MeV software, highlighting four biological pathways. Genes with the most significant expression changes are indicated. (**D**) Kyoto Encyclopedia of Genes and Genomes (KEGG) pathway analysis showing genes involved in the pyruvate metabolic pathway, PTS genes, and TCS-related genes.

RNA-seq analysis revealed significant differences in gene expression between the WT and SMU.1147 deletion mutant, supporting the hypothesis that the SMU.1147 gene influences the expression of other genes, including those involved in competence. Genes showing more than a twofold difference in expression were categorized as significantly upregulated or downregulated, with the results summarized in Tables S1 and S2. A volcano plot (Fig. 1A) highlighted 79 genes with more than a twofold increase (log_2_ SMU.1147/WT ratio ≥ 1, *P* < 0.05) and 270 genes with more than a twofold decrease (log_2_ SMU.1147/WT ratio ≤ −1, *P* < 0.05).

Gene ontology and pathway analyses using the Database for Annotation, Visualization, and Integrated Discovery (DAVID) program (https://david.ncifcrf.gov/) showed that differently expressed genes (DEGs) were enriched in pathways related to cell interactions, defense mechanisms, and transport (EASE score < 0.05; Fig. 1B). Notably, genes associated with stress responses and metabolic adaptations showed significant changes. Heatmap analysis of the genes commonly involved in each pathway further underscored these findings (Fig. 1C). For example, the three most upregulated genes (SMU.1148, SMU.1149, and SMU.1150, also named *lctFEG*) encode an ABC transporter, previously linked to resistance against the lantibiotic nukacin ISK-1 (30). In contrast, *spxA2*, a gene crucial for oxidative stress resistance, growth, and antibiotic tolerance, was among the most downregulated genes (31–33). Additionally, the *dnaK* operon (*hrcA-grpE-dnaK*), which plays a role in the heat shock response and adaptation to low pH (34), was upregulated in the mutant, suggesting an adaptive stress response.

Kyoto Encyclopedia of Genes and Genomes (KEGG) pathway analysis highlighted alterations in genes associated with the TCSs and quorum-sensing pathways, both essential for virulence and stress resistance. Significant downregulation of key stress tolerance- and virulence-associated genes, such as *htrA*, *dnaA*, and *ciaXRH* (35, 36), was observed, potentially contributing to the competence defects seen in the mutant. In contrast, pyruvate metabolism-related genes (*adhA*, *adhB*, *adhC*, *adhD*, *pdhC*, *pdhD*, *accA*, *accC*, and *accD*) were upregulated, indicating a shift in metabolic regulation in the absence of SMU.1147 (Fig. 1D). Collectively, these data suggest that SMU.1147 modulates genes critical for stress tolerance, pathogenicity, and metabolic processes, thereby influencing key aspects of *S. mutans* physiology and pathogenesis.

### Impact of SMU.1147 mutation on genes involved in cellular metabolism and cell processes

**Fig 2:**
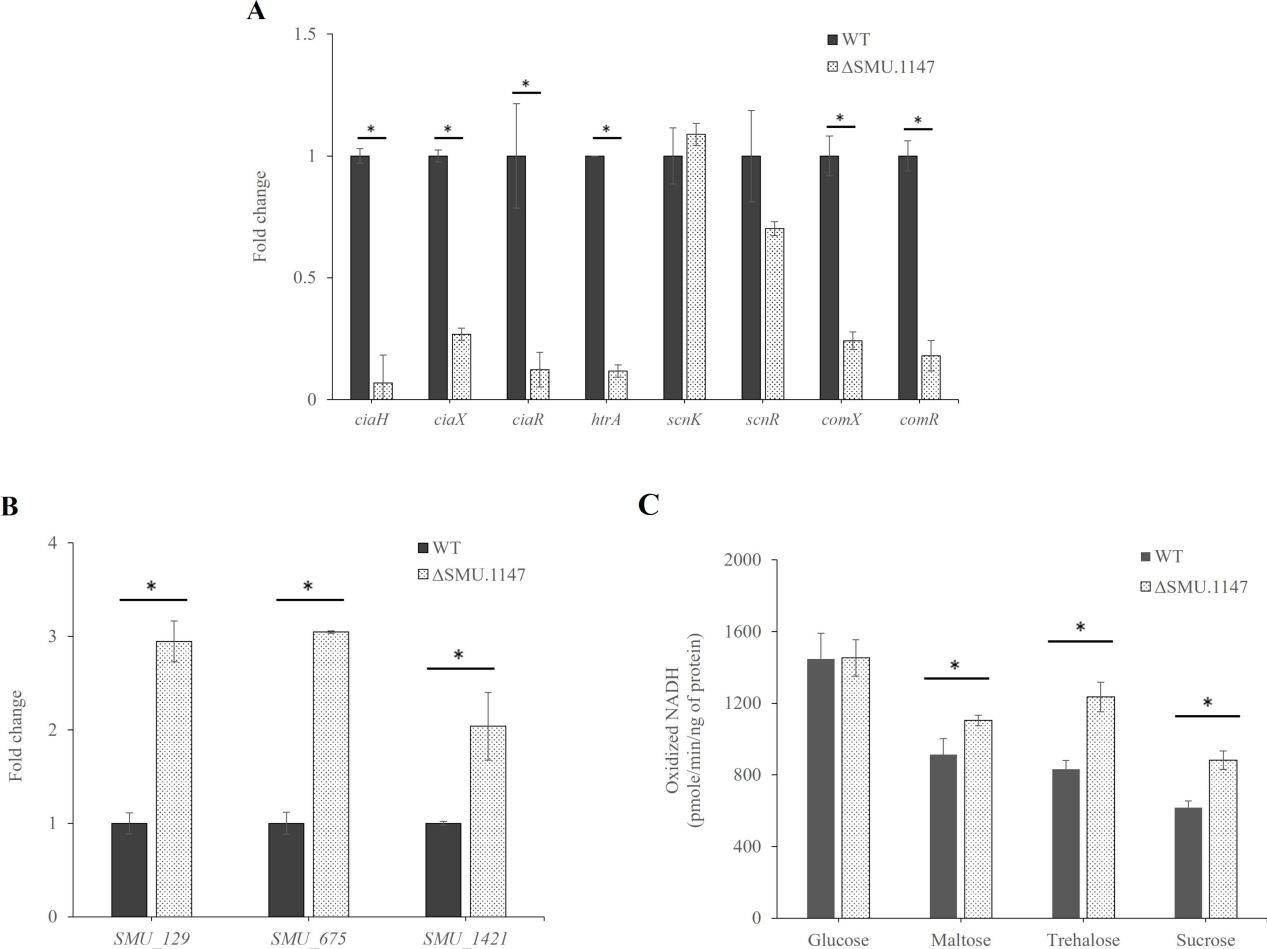
Effects of SMU.1147 mutation on gene expression and carbohydrate intake. Expression levels of eight targets (**A**) and genes involved in glycogen metabolism (**B**) were compared using qRT-PCR. Data indicate fold changes of target RNA derived from 1 µg of input RNA after normalization to 16S rRNA levels in the same samples. (**C**) PTS transport of glucose, maltose, trehalose, or sucrose was determined using permeabilized cells as described in the Materials and Methods section. An asterisk (*) indicates a significant difference from the WT genetic background at *P* < 0.05. Results are expressed as mean values from three biological repeats, with error bars representing SDs.

The significant downregulation of *ciaXHR* (3.7-, 14.6-, and 8.1-fold, respectively) and *htrA* (8.5-fold) in the SMU.1147 mutant aligns with the proposed role of SMU.1147 in modulating stress responses and genetic competence (Fig. 2A). Similarly, *comX* and *comR* showed reductions of 4.1- and 5.5-fold, respectively, as confirmed by quantitative real-time PCR (qRT-PCR). Although these genes did not exhibit statistically significant changes in the RNA-seq data (0.78-fold, *P* = 0.32; 0.60-fold, *P* = 0.01, respectively), these genes were prioritized for validation due to their central roles in competence regulation and quorum-sensing pathways. Interestingly, the expression levels of *scnK* (SMU.1145) and *scnR* (SMU.1146), located in the same operon as SMU.1147, remained unchanged, suggesting that SMU.1147 selectively regulates downstream genes rather than broadly affecting the operon. These findings highlight the critical role of SMU.1147 in modulating peptide-mediated signaling pathways, including CSP- and XIP-dependent systems, and its specific impact on stress response and competence-related genes.

The upregulation of enzyme I (EI, encoded by *ptsI*), along with genes involved in maltose and trehalose metabolism (*malT* and *treB*, respectively), indicates an increased capacity for sugar uptake and phosphorylation in the SMU.1147 mutant (Fig. 2B). The inclusion of SMU.1421 in the qRT-PCR analysis was guided by KEGG pathway analysis, which identified it as a key gene involved in the conversion of pyruvate to acetyl-CoA (Fig. 1D). While RNA-seq data showed a 2.468-fold increase in SMU.1421 expression in the mutant, this change did not reach statistical significance (*P* = 0.15). Given its metabolic implications, particularly in acetoin production through the acetolactate pathway, SMU.1421 was further validated using qRT-PCR. This was corroborated by PTS assays (Fig. 2C), which showed an increase in the uptake of maltose, trehalose, and sucrose in the mutant strain. However, despite increased sugar uptake, the growth rate and ATP production showed no significant differences (data not shown), suggesting that while the mutant efficiently imports sugars, it struggles to convert them into energy, potentially due to downstream inefficiencies in carbohydrate metabolism.

### Effect of SMU.1147 mutation on organic acid production

**Fig 3:**
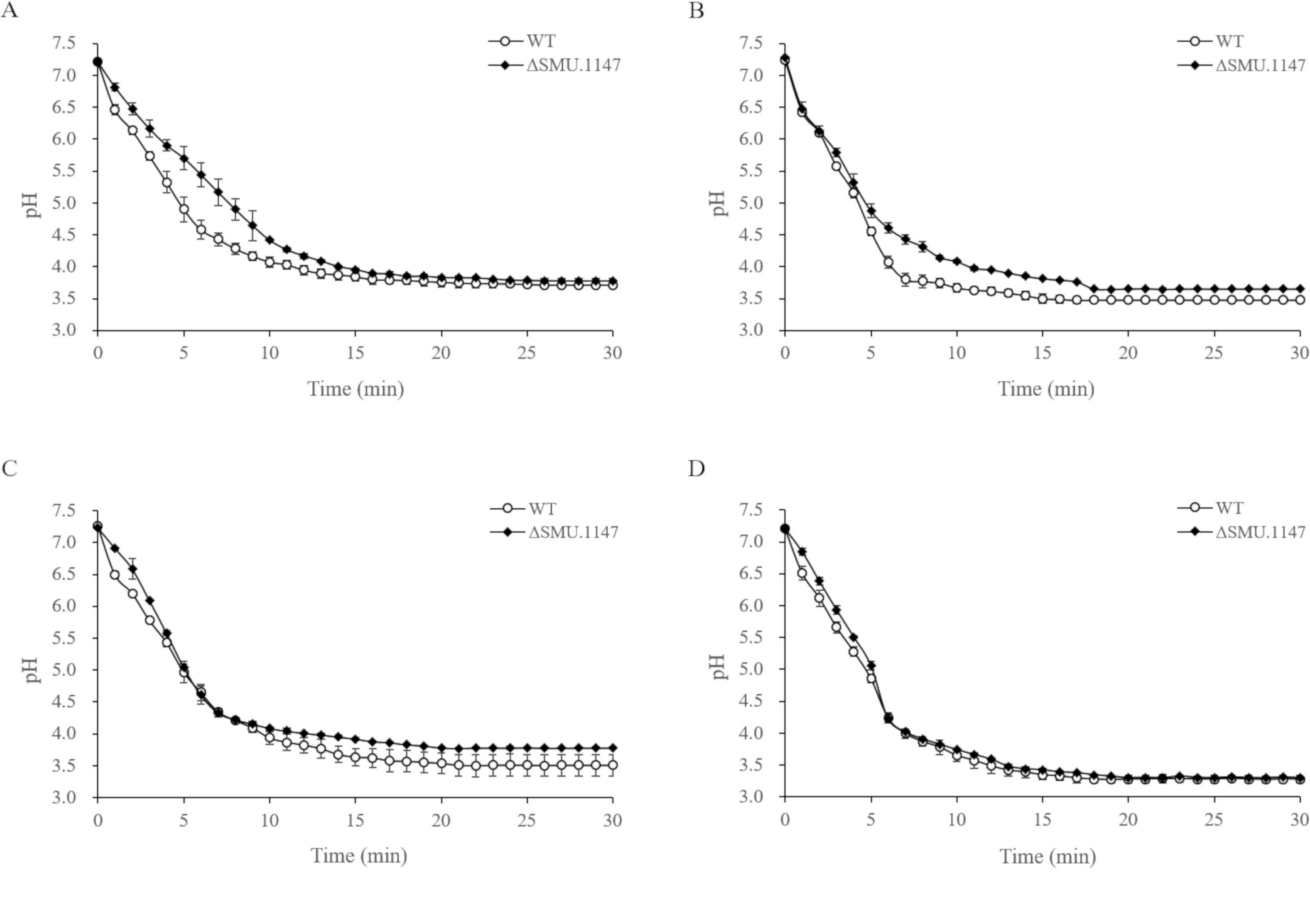
Acidogenicity of *S. mutans* WT and SMU.1147 mutant strains. *S. mutans* WT and SMU.1147 strains were cultured to mid-exponential phase in brain heart infusion (BHI) medium supplemented with 20 mM glucose. The assay was initiated by adding 1 M of (**A**) glucose, (**B**) maltose, (**C**) trehalose, and (**D**) sucrose. pH decrease was monitored every minute for 30 min. Data represent averages from three independent experiments. Symbols (closed diamonds and open circles) indicate *S. mutans* wild-type and mutant strains, respectively, with error bars representing SDs.

The pH measurements of the growth medium indicated that, despite the upregulation of sugar metabolism genes, the SMU.1147 mutant produced fewer organic acids compared to the WT strain. The final pH values were consistently higher in the mutant, indicating reduced acid production (Fig. 3). When cells were grown on glucose or maltose, the WT strain exhibited a faster and more pronounced pH drop (Fig. 3A and B; *P* < 0.05), demonstrating its superior ability to convert sugars into organic acids.

In the presence of trehalose, the final pH was 3.51 ± 0.17 for the WT strain and 3.78 ± 0.02 for the mutant (Fig. 3C; *P* < 0.05), suggesting reduced efficiency of the mutant in metabolizing trehalose. No significant difference in pH was observed in sucrose-containing media (Fig. 3D), suggesting that sucrose metabolism was less affected by SMU.1147 deletion. These findings suggest a metabolic shift in the mutant strain that is likely linked to the observed changes in gene expression, where carbon flux toward organic acid production is reduced, possibly due to disruptions in glycolytic or fermentation pathways.

### Effects on cell growth and survival

**Fig 4:**
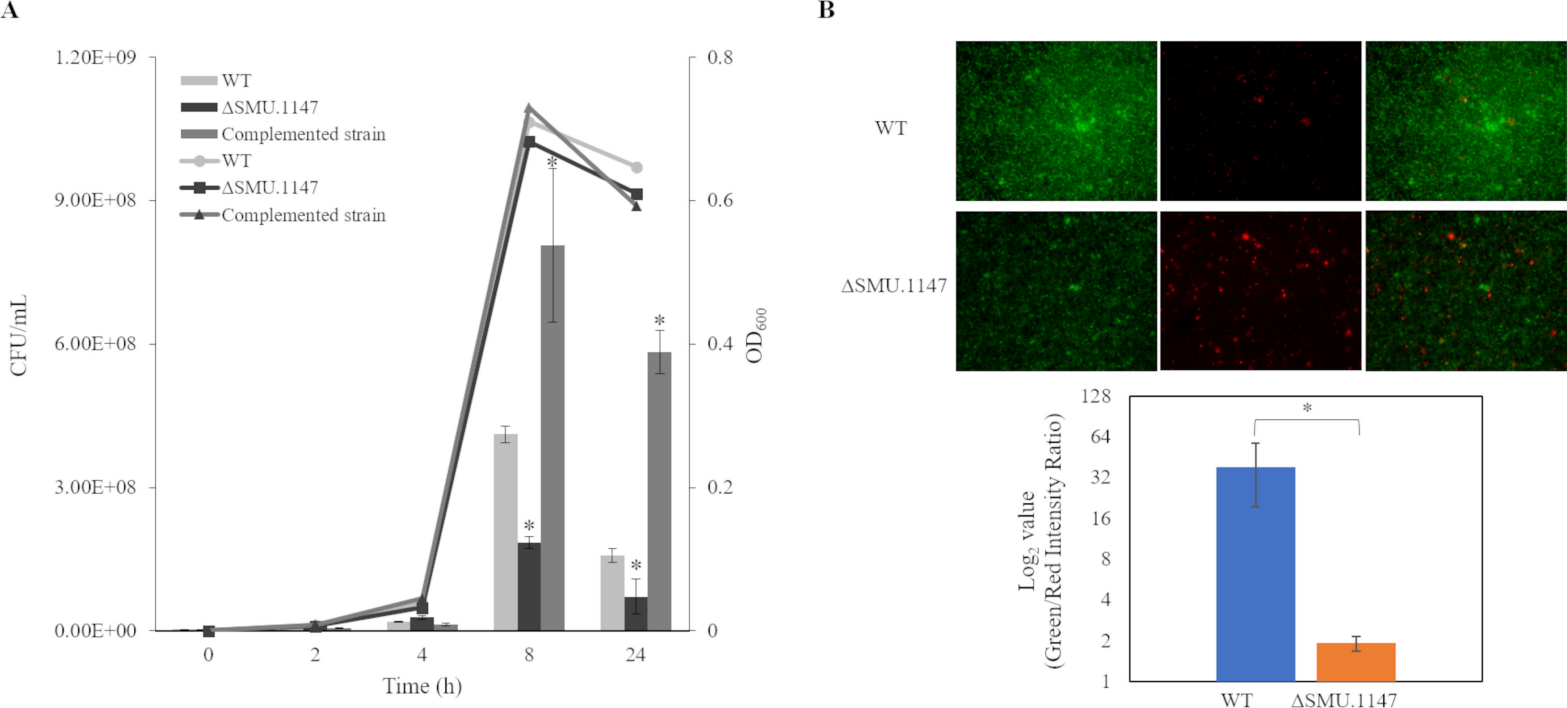
Comparison of cell survival between *S. mutans* WT and mutant strains. (**A**) Growth curves (lines) showed no significant differences among the WT, ΔSMU.1147 mutant, and complemented strains. Colony counts (bars) at various time points (0, 2, 4, 8, and 24 h) were conducted for the WT, ΔSMU.1147 mutant, and complemented strains for comparison. Results are expressed as mean values from three biological repeats, with error bars representing SDs. (**B**) *S. mutans* WT and mutant strains were grown to OD_600_ 0.3 in BHI broth. Cells were observed using a fluorescent microscope, after staining using LIVE/DEAD BacLight Bacterial Viability Kit. Cells were observed under a fluorescence microscope, and the green-to-red fluorescence intensity ratio was calculated using ImageJ software (https://imagej.net) to assess cell viability. An asterisk (*) indicates a significant difference from the WT genetic background at *P* < 0.05.

Although no significant differences were observed in growth rates between the WT and mutant strains in brain heart infusion (BHI) medium (Fig. 4A, lines), the colony formation assays revealed a marked reduction in viable cells for the SMU.1147 mutant at both 8 and 24 h time points (Fig. 4A, bars; *P* < 0.05). Notably, the complemented strain, in which a copy of the SMU.1147 gene was expressed from the pIB184 plasmid, restored colony-forming units (CFU) counts to WT levels, confirming that the observed defect is specifically associated with the deletion of SMU.1147. These findings suggest that, despite normal growth rates, the SMU.1147 mutant experiences metabolic stress, leading to cell death over time.

Further analysis using LIVE/DEAD staining at the 16 h time point confirmed increased cell death in the mutant strain, as reflected by a significantly lower intensity ratio of green-to-red fluorescence (1.93 ± 0.24) compared to the WT (38.57 ± 19.00; Fig. 4B; *P* < 0.05). This indicates that while capable of normal growth, the mutant suffers from an inability to maintain long-term viability. These findings highlight the impact of SMU.1147 deletion on long-term survival, particularly in nutrient-rich conditions, where the mutant strain appears to mismanage its metabolic resources despite unchanged ATP levels.

Interestingly, these results contrast with previous studies in a chemically defined medium(FMC), where the SMU.1147 mutant exhibited altered growth rates (Fig. 4A) (24), suggesting that the impact of the SMU.1147 deletion on growth is highly dependent on the metabolic environment. Complex media, such as BHI, may obscure certain metabolic deficiencies, whereas defined media highlight the metabolic vulnerabilities of this mutant. These findings reinforce the idea that SMU.1147 is critical for metabolic flexibility and survival under diverse environmental conditions.

## DISCUSSION

**Fig 5:**
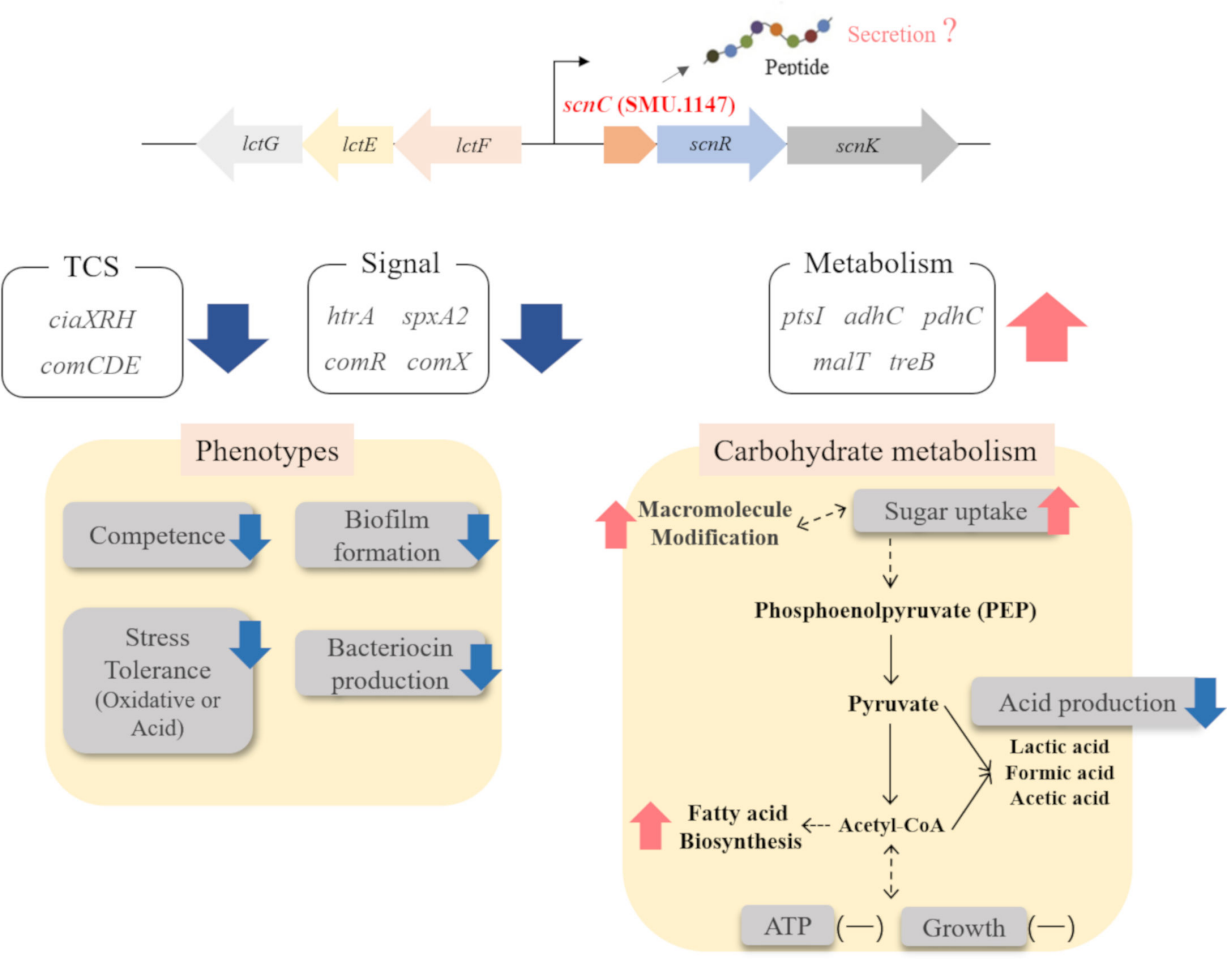
Schematic diagram for the role of SMU.1147 in *S. mutans*. The deletion of SMU.1147 affects phenotypes related to competence, biofilm formation, and stress tolerance. Transcriptome analysis reveals its impact on the expression of genes related to TCSs and other key genes such as *htrA* and *spxA2*. Increased sugar uptake was not correlated with organic acid production, ATP levels, or growth. The proposed pathway indicates possible energy utilization routes, including macromolecule modifications such as serotype-specific carbohydrate antigens. Red and blue marks indicate increased and decreased expression, respectively, and (-) marks denote no significant changes.

The oral environment is subject to continual fluctuations, necessitating rapid adaptation for the survival of *S. mutans*. This adaptive capability has evolved over the centuries through cumulative trait variations, enabling *S. mutans* to withstand diverse human dietary habits and modern lifestyles. SMU.1147, a unique core gene of *S. mutans*, plays a significant role in modulating biofilm formation, stress response to external stimuli, and genetic competence (24). In the present study, transcriptome analysis revealed that SMU.1147 influenced key pathways involved in carbohydrate metabolism, stress response, and quorum sensing, highlighting its importance in the pathogenic potential of this bacterium, as summarized in Fig. 5.

Transcriptome analysis showed that the deletion of SMU.1147 led to a significant upregulation of genes involved in sugar metabolism and stress management, particularly the *lctFEG* ABC transporter cluster (SMU.1148–1150), which plays a role in bacteriocin immunity (30). This observation aligns with the known function of the LctFEG transporter in exporting nukacin ISK-1, a lantibiotic produced by *Staphylococcus warneri*, suggesting that the upregulation of *lctFEG* in the SMU.1147 deletion mutant may serve as a compensatory mechanism to protect the cell from external threats such as bacteriocins. SMU.1147 is a part of the ScnRK (also designated as LcrRS) TCS, which regulates the expression of *lctFEG*. Previous studies have shown that the ScnRK system responds to nukacin ISK-1 by upregulating the *lctFEG* transporter, which likely exports nukacin ISK-1 from cells (30). Thus, our findings suggest that the absence of SMU.1147 may dysregulate this system, leading to an increased expression of *lctFEG*.

The increased expression of several PTS transporters, particularly those related to maltose and trehalose uptake, suggests that the mutant strain enhanced sugar intake to compensate for the loss of SMU.1147. Furthermore, the significant upregulation of genes involved in pyruvate metabolism, such as SMU.129, SMU.675, and SMU.1421, suggests that the absence of SMU.1147 affects the pathways critical for energy production and stress defense (37, 38). These metabolic adjustments appeared to be attempts by the mutant strain to manage energy inefficiencies and maintain viability under stress. However, despite this upregulation, the mutant did not exhibit increased ATP production and showed slightly reduced efficiency of organic acid production when grown in media supplemented with these sugars, indicating impaired carbon flow regulation. These findings imply that the absence of SMU.1147 disrupts the coordination of energy metabolism, possibly leading to a shift in metabolic priorities toward stress management. However, consistent with the previous report (24), the significant reduction in cell viability under stress conditions, even after prolonged incubation, suggests that compensatory mechanisms may not be fully effective.

The SMU.1147 deletion strain also exhibited a lower growth rate under acidic or oxidative stress conditions when grown in an FMC medium than the WT strain (24). This reduction may stem from the dysregulated expression of stress-related genes, particularly *spxA2*, which is vital for oxidative stress tolerance (39). SpxA2, along with its paralog SpxA1, helps maintain the redox balance and protein quality control through its interaction with the ClpP proteolytic system (32, 39). Our transcriptomic data revealed a notable decrease in *spxA2* expression in the mutant, corresponding to impaired stress responses and reduced growth under FMC conditions, in which the bacteria were more susceptible to environmental stressors. However, our growth measurements in the BHI medium showed no significant changes, as noted in previous studies. Thus, the relationship between SpxA2 and SMU.1147 underscores the critical role of SMU.1147 in modulating oxidative and acid stress responses, which are essential for optimal growth under nutrient-limited conditions. The reduced expression of *clpP* and *clpX* observed in the SMU.1147 deletion mutant supported this hypothesis. These genes are crucial for the degradation of misfolded or damaged proteins during stress conditions, and their downregulation suggests a compromised ability to manage protein quality, particularly under oxidative stress (39, 40). Impaired protein homeostasis can lead to the accumulation of damaged proteins, further reducing bacterial fitness. Moreover, Spx proteins have been shown to regulate genes involved in biofilm formation, and their reduced expression in the mutant strain may contribute to the observed growth defects by limiting biofilm development, which is a key survival strategy for *S. mutans* in fluctuating environments.

In addition to its effects on metabolic pathways, the inactivation of SMU.1147 significantly affected genetic competence mechanisms. Notably, the expression of key genes such as *htrA* and *ciaRH*, which are involved in thermal stress defense and biofilm formation (35, 36), was reduced in the mutant strain. In particular, HtrA is regulated by the CiaRH TCS, which modulates competence development and defense responses in *S. mutans* (35, 36). HtrA activation involves CiaR, a central component of the CiaRH TCS. The interplay among the CiaRH system, HtrA, and SMU.1147 can be compared to the relationship between the ComDE system and competence regulation in *S. mutans*. Similar to ComDE, which activates competence through the accumulation of extracellular CSP, the CiaRH system functions as a negative regulator of competence and indirectly influences competence gene expression via HtrA (35, 41, 42). HtrA, which is upregulated by CiaR, not only ensures protein quality control under stress but also disrupts competence processes by degrading components related to quorum sensing (43), such as the peptide pheromone BlpC in *Streptococcus pneumoniae* (44). In this regulatory framework, SMU.1147 serves as a critical integrator that links metabolic and stress response pathways to both the CiaRH and ComDE systems. In this regulatory framework, SMU.1147 serves as a critical integrator that links metabolic and stress response pathways to both the CiaRH and ComDE systems. Although the downregulation of *ciaRH* and *htrA* in the SMU.1147 mutant suggests relief from the negative regulation of competence, the expected increase in competence was not observed. This paradox may be due to the impaired stress response in the mutant strain, which limits the ability of cells to utilize the reduced inhibition by CiaRH. The compromised stress defenses could hinder overall cellular fitness, preventing the mutant from achieving the expected enhancement in competence despite the downregulation of *ciaRH* and *htrA*. Thus, the downregulation of both *htrA* and *ciaRH* in the SMU.1147 mutant indicates a disruption in the stress pathways, compromising bacterial survival.

Furthermore, the involvement of SMU.1147 in the ScnRK TCS presents a potentially novel regulatory pathway for *S. mutans*. Although the ComCDE and ComRS pathways have traditionally been implicated in the regulation of genetic competence and stress responses, our findings suggest that SMU.1147 contributes to an independent signaling mechanism. The SMU.1147-derived peptide acts as an extracellular signal within the ScnRK system in response to environmental stimuli such as bacteriocins. This peptide likely influences the ScnRK TCS, allowing the bacterium to integrate environmental cues and modulate its genetic competence. Importantly, this regulatory mechanism appeared to operate separately from the CSP and XIP pathways, which are conventionally involved in the regulation of competence in *S. mutans* (45). By transmitting distinct signals through ScnRK, SMU.1147 adds complexity to the regulatory network and provides *S. mutans* with a broader ability to adapt to various environmental conditions. This novel signaling route suggests that *S. mutans* may use multiple overlapping systems to finely tune its response to external stressors, thereby enhancing its survival and competitiveness in complex microbial communities. Thus, our findings suggest a new model in which SMU.1147 participates in a unique peptide-mediated pathway (24, 45), offering further insight into how *S. mutans* processes environmental signals to regulate competence and stress adaptation.

In conclusion, SMU.1147 appears to act as a key regulator of both the metabolic and stress response pathways in *S. mutans*, influencing its ability to adapt to environmental fluctuations and maintain its pathogenic potential. The upregulation of carbohydrate transport systems and stress regulon genes in the SMU.1147 mutant emphasizes its role in modulating metabolic homeostasis and defense mechanisms. These findings open up avenues for further research into the precise molecular mechanisms by which SMU.1147 interacts with other regulatory systems, including TCSs, to control the virulence and adaptability of *S. mutans* in the oral cavity.

## MATERIALS AND METHODS

### Strains, culture conditions, and growth measurement

The wild-type (WT) strain *S. mutans* UA159, SMU.1147 deletion mutant, and complement strains were used as listed in Table S3. *Escherichia coli* DH10B served as the host for the shuttle vector. *S. mutans* strains were routinely grown in BHI media (BD Bioscience, New Jersey, USA) at 37°C under 5% CO_2_ conditions. *E. coli* strains were inoculated onto Luria-Bertani medium (BD Biosciences) and cultured at 37°C under aerobic conditions. Antibiotics were supplemented if required; kanamycin (1 mg/mL for *S. mutans*; Sigma-Aldrich, St. Louis, USA), spectinomycin (1 mg/mL; Duchefa, Haarlem, Netherlands), erythromycin (300 µg/mL for *E. coli*; Sigma-Aldrich), and spectinomycin (100 µg/mL for *E. coli*; Duchefa).

For growth experiments, *S. mutans* WT and mutant strains were first grown in a BHI medium at 37°C with 5% CO_2_ for 16 h (46). The cultures were diluted 1:100 in fresh BHI medium and incubated until the early exponential phase (OD_600_ = 0.2–0.3). For further analysis, 300 µL of fresh BHI or tryptone-vitamin (TV) liquid medium was added to each well of a honeycomb plate (Oy Growth Curves Ab Ltd., Helsinki, Finland), and 3 µL of the previously cultured inoculum was added to each well. Growth was monitored by measuring absorbance at 600 nm every hour at 37°C using a Bioscreen C plate reader (Oy Growth Curves Ab Ltd) for 24 h. To examine growth in the presence of different carbohydrates, the TV medium was modified by supplementing it with individual carbohydrates (glucose, sucrose, maltose, or trehalose), each at a final concentration of 20 mM. Mineral oil was layered on the top to create oxygen-limited conditions.

### RNA isolation, transcriptome analysis, and qRT-PCR

Cells were cultivated to the late exponential phase (OD_600_ = 0.6–0.7) in BHI media at 37°C under 5% CO_2_. Cell harvesting was performed by centrifugation at 4°C and 18,000 × *g* for 10 min. To stabilize the recovered cells, 1 mL of bacterial RNA protect reagent (Qiagen, Hilden, Germany) was added to the pellet, and the mixture was allowed to react at room temperature for 5 min. Cells were collected by centrifugation at 4°C and 18,000 × *g* for 10 min and resuspended in 50/10 mM TE buffer (50 mM Tris, 10 mM EDTA, and pH 7.5) containing SDS. The resuspended cells were then added to a 2 mL screw cap tube containing 300 µL of cold acid phenol and 250 µL of glass beads. The cells were disrupted twice by bead beating using a bead beater (Biospec Products, Inc., Bartlesville, OK, USA) at 4°C. Total RNA was isolated using the RNeasy Mini Plus Kit (Qiagen), following the manufacturer’s instructions. RNA concentration was determined using a Nanodrop Spectrophotometer (Thermo Fisher Scientific, Waltham, MA, USA).

RNA-seq analysis involved paired-end sequencing on a NovaSeq 6000, conducted by a specialized service provider (Ebiogen, Seoul, South Korea). The results were analyzed based on the reference genome of the NCBI RefSeq assembly (ASM746v2, *S. mutans* UA159), with details summarized in Table S4. DEGs were analyzed using the ExDEGA program (Ebiogen), and functional annotation was performed using the DAVID program. Heatmap and KEGG analysis were used for pathway analyses. Through these analyses, DEGs between the WT and mutant strains were identified and are listed in Tables S1 and S2.

One microgram of RNA was used to synthesize cDNA using the PrimeScript RT Reagent Kit (Takara, Shiga, Japan) with random hexamers. The PCR mixture was prepared using gene-specific primers for individual targets (Table S3) and RbTaq qPCR 2 × Premix (SYBR Green with high ROX; Enzynomics, Daejeon, South Korea). Real-time amplification was conducted in the StepOnePlus Real-Time PCR system (Applied Biosystems, Waltham, MA, USA) through an initial denaturation step at 95°C for 3 min, followed by 40 cycles: denaturation at 95°C for 30 s, annealing at 60°C for 30 s, and extension at 72°C for 30 s. All experiments were performed in triplicate, and the results were normalized to the amplification of the 16S rRNA gene, which served as an endogenous control. The 2^−∆∆Ct^ method was employed for relative quantitative analysis, and the results were presented as mean values with SDs.

### Phosphoenolpyruvate-dependent PTS assay

Phosphoenolpyruvate (PEP)-dependent transport activities were determined using a PTS assay as described previously (47). Briefly, cells grown to the early exponential phase were inoculated into TV medium supplemented with individual carbohydrates (glucose, sucrose, maltose, or trehalose) at a final concentration of 20 mM. The cultures were allowed to reach an OD_600_ value of 0.5–0.6, and the cells were harvested via centrifugation. Cells were washed twice with 0.1 M sodium potassium phosphate buffer (NaKPO_4_) containing 5 mM MgCl_2_ (pH 7.2) and resuspended in 0.1 vol of the same buffer. The resuspended cells were permeabilized with a toluene-acetone (1:9) solution by vortexing twice at 2 min intervals. The reaction mix was formulated by incorporating 0.05 mM NADH, five units of lactate dehydrogenase, and 50 mM specific carbohydrate into permeabilized cells. The reaction was initiated by the addition of 2.5 mM PEP, and the rate of NADH oxidation was measured at 340 nm using a Multiskan FC microplate reader (Thermo Fisher Scientific). PTS activity was normalized by protein concentration, which was measured using a bicinchoninic acid kit (Thermo Fisher Scientific) (48).

### pH drop assay

Acid-end product production was assessed using a pH drop assay, as previously described (49). The cells were cultured in a BHI medium for 16 h, followed by a 1:20 dilution in 50 mL of fresh BHI medium. The cells were allowed to reach an OD_600_ value of 0.5–0.6 and harvested via centrifugation at 18,000 × *g* at 4°C for 5 min. After washing the pellets twice with ice-cold distilled water at 4°C, the cells were resuspended in a solution containing 5 mL of 50 mM KCl and 5 mM MgCl_2_. The pH was adjusted to 7.2 by the addition of 1 µL of 0.1 M KOH. Next, 250 µL of the carbohydrate source tested was introduced for stabilization, and pH changes were monitored every minute for 30 min using a SevenCompact pH meter S220 (Mettler-Toledo, Columbus, OH, USA).

### Cell viability assay

Cells were cultured in a BHI medium at 37°C under 5% CO_2_ for 16 h. The cultures were then diluted 1:100 in fresh BHI medium and incubated at 37°C under 5% CO_2_ until reaching the early exponential phase (OD_600_ = 0.2–0.3). To determine the number of live bacterial cells, cultures at each time point (0, 2, 4, 8, and 24 h) were serially diluted and plated onto a BHI medium. Live cell counts were determined in CFU/mL. The confirmation was made that live/dead cells were stained with the LIVE/DEAD BacLight Bacterial Viability Kit (Thermo Fisher Scientific) and visualized using a Zeiss Axio Observer 3 inverted microscope (Carl Zeiss, Oberkochen, Germany) at 24 h.

### Transformation measurements

The transformation efficiency was measured as described previously (50). Briefly, overnight cultures were diluted (1:20) with 450 µL of fresh BHI medium and 20 µL of horse serum and further incubated for 2 h and 30 min at 37°C under 5% CO_2_. The cultures were supplemented with synthetic CSPs at a final concentration of 400 nM and incubated for 30 min. After the incubation, the cultures were introduced to the pDL278 plasmid containing the spectinomycin resistance gene (Sp^r^) at a final concentration of 400 ng and incubated anaerobically for 3 h. The resulting cultures were serially diluted, and the transformation efficiency was measured on spectinomycin plates in colony-forming units per milliliter. The total number of colonies on non-selective BHI plates was measured to adjust the cell count.

### Statistical analysis

All experiments were conducted independently and repeated at least in triplicate, following the same procedure. One-way analysis of variance and Student’s *t*-test were used to analyze the data, with statistical significance confirmed at a 95% confidence level (*P* < 0.05). In all cases, *P* values ≤ 0.05 were considered statistically significant.

## Data Availability

The raw RNAseq data generated in this study have been submitted to the Gene Expression Omnibus (GEO) database and are publicly available under accession number GSE244268 (GEO Accession viewer).

## SUPPLEMENTAL MATERIAL

Supplemental material spectrum.02917-24-s0001.docx Tables S1 to S4.

ASM does not own the copyrights to Supplemental Material that may be linked to, or accessed through, an article. The authors have granted ASM a non-exclusive, world-wide license to publish the Supplemental Material files. Please contact the corresponding author directly for reuse.
