# Supplementary material for: Deciphering the role of SMU.1147 in peptide-mediated signaling and competence in Streptococcus mutans
Source: Microbiol Spectr. 2025 Mar 5;13(4):e02917-24. doi: 10.1128/spectrum.02917-24 (PMC11960140; doi:10.1128/spectrum.02917-24)
Supplement: Supplemental material — Tables S1 to S4. [file spectrum.02917-24-s0001.docx]

[Supplemental materials]

Deciphering the Role of SMU.1147 in Peptide-Mediated Signaling and Competence in *Streptococcus mutans*

Jeong Nam Kim^1,2*^, Si-Uk Ryoo^1^ and Yeuna Nam^1^

^1^ Department of Integrated Biological Science, Pusan National University, Busan 46241, Korea

^2^ Department of Microbiology, College of Natural Sciences, Pusan National University, Busan 46241, Korea

^*^Corresponding author:

Department of Microbiology

Pusan National University, College of Natural Sciences

Advanced Science building 609, Busandaehang-ro,

63beon-gil, Geumjeong-gu,

Busan 46241, South Korea

Phone: 82-51-510-2269

Fax: 82-51-514-1778

E-mail: [kimjn@pusan.ac.kr](mailto:kimjn@pusan.ac.kr)

**Table S1.** **Up-regulated genes in DEG dataset.**

| Locus Tag | Gene name | Fold change | Description |
| --- | --- | --- | --- |
| SMU.48 | *purD* | 2.128 | phosphoribosylamine--glycine ligase |
| SMU.50 | *purE* | 2.084 | 5-(carboxyamino)imidazole ribonucleotide  mutase |
| SMU.89c |  | 2.527 | formate/nitrite transporter family protein |
| SMU.119 |  | 2.611 | S-(hydroxymethyl)glutathione dehydrogenase  /class III alcohol dehydrogenase |
| SMU.128 |  | 2.154 | alpha-ketoacid dehydrogenase subunit beta |
| SMU.129 |  | 2.301 | dihydrolipoamide acetyltransferase |
| SMU.130 | *lpdA* | 2.229 | dihydrolipoyl dehydrogenase |
| SMU.130 | *lpdA* | 2.488 | dihydrolipoyl dehydrogenase |
| SMU.131 |  | 2.497 | lipoate--protein ligase |
| SMU.132 |  | 2.365 | amidohydrolase |
| SMU.155 | *pnp* | 2.100 | polyribonucleotide nucleotidyltransferase |
| SMU.179 |  | 3.554 | NAD(P)H-dependent oxidoreductase |
| SMU.247 | *sufC* | 2.142 | Fe-S cluster assembly ATPase SufC |
| SMU.248 | *sufD* | 2.288 | Fe-S cluster assembly protein SufD |
| SMU.249 |  | 2.085 | cysteine desulfurase |
| SMU.273 |  | 2.091 | 3-keto-L-gulonate-6-phosphate decarboxylase  UlaD |
| SMU.275 |  | 2.176 | L-ribulose-5-phosphate 4-epimerase |
| SMU.297 | *polA* | 2.786 | DNA polymerase I |
| SMU.298 |  | 2.858 | CoA-binding protein |
| SMU.303 |  | 2.224 | MBL fold metallo-hydrolase |
| SMU.304 | *tadA* | 2.470 | tRNA adenosine(34) deaminase TadA |
| SMU.305 |  | 2.270 | hypothetical protein |
| SMU.348 |  | 2.391 | HIT family protein |
| SMU.385 | *tsaB* | 2.483 | tRNA (adenosine(37)-N6)-  threonylcarbamoyltransferase complex  dimerization subunit type 1 TsaB |
| SMU.527 |  | 2.381 | dihydrodipicolinate reductase |
| SMU.528c |  | 2.100 | antibiotic biosynthesis monooxygenase |
| SMU.577 | *lytS* | 3.194 | two-component system sensor histidine kinase LytS |
| SMU.598 | *recR* | 2.374 | recombination mediator RecR |
| SMU.666 |  | 2.658 | acetylornithine transaminase |
| SMU.668c | *nrdE* | 2.315 | class 1b ribonucleoside-diphosphate reductase  subunit alpha |
| SMU.675 | *ptsI* | 2.775 | phosphoenolpyruvate—protein phosphotransferase |
| SMU.746c |  | 2.746 | TIGR03943 family protein |
| SMU.747c |  | 3.000 | permease |
| SMU.765 | *ahpF* | 2.141 | alkyl hydroperoxide reductase subunit F |
| SMU.841 |  | 2.155 | cysteine desulfurase |
| SMU.842 | *thiI* | 2.134 | tRNA 4-thiouridine(8) synthase ThiI |
| SMU.853 | *lspA* | 2.223 | signal peptidase II |
| SMU.854 |  | 2.203 | RluA family pseudouridine synthase |
| SMU.855 |  | 2.006 | TVP38/TMEM64 family protein |
| SMU.880 |  | 2.033 | carbohydrate ABC transporter permease |
| SMU.942 |  | 2.497 | hydroxymethylglutaryl-CoA reductase,  degradative |
| SMU.1148 |  | 8.114 | ABC transporter ATP-binding protein |
| SMU.1149 |  | 6.004 | ABC transporter permease |
| SMU.1150 |  | 7.155 | ABC transporter permease |
| SMU.1182 |  | 2.242 | mannitol-1-phosphate 5-dehydrogenase |
| SMU.1222 | *pyrF* | 2.261 | orotidine-5'-phosphate decarboxylase |
| SMU.1235 | *mnmE* | 2.047 | tRNA uridine-5-  carboxymethylaminomethyl(34) synthesis  GTPase MnmE |
| SMU.1239 | *pepV* | 2.834 | dipeptidase PepV |
| SMU.1252 |  | 2.013 | glycerate kinase |
| SMU.1293 | *sufB* | 2.969 | Fe-S cluster assembly protein SufB |
| SMU.1322 |  | 2.267 | (S)-acetoin forming diacetyl reductase |
| SMU.1323 |  | 2.051 | MBL fold metallo-hydrolase |
| SMU.1389 |  | 2.677 | phosphoenolpyruvate carboxykinase (ATP) |
| SMU.1410 |  | 2.077 | FAD-dependent oxidoreductase |
| SMU.1425 |  | 2.100 | AAA family ATPase |
| SMU.1561 |  | 3.373 | NAD-binding protein |
| SMU.1562 |  | 3.736 | NAD-binding protein |
| SMU.1563 |  | 3.563 | cation-transporting P-type ATPase |
| SMU.1612c |  | 2.291 | class I SAM-dependent methyltransferase |
| SMU.1613c | *coaE* | 2.025 | dephospho-CoA kinase |
| SMU.1614 | *mutM* | 2.262 | DNA-formamidopyrimidine glycosylase |
| SMU.1615c |  | 2.219 | DUF664 domain-containing protein |
| SMU.1616c |  | 2.170 | NUDIX hydrolase |
| SMU.1620 |  | 2.091 | PhoH family protein |
| SMU.1676c |  | 2.018 | polysaccharide biosynthesis protein |
| SMU.1736 | *accC* | 2.060 | acetyl-CoA carboxylase biotin carboxylase  subunit |
| SMU.1737 | *fabZ* | 2.048 | 3-hydroxyacyl-ACP dehydratase FabZ |
| SMU.1851 | *uvrA* | 2.334 | excinuclease ABC subunit UvrA |
| SMU.187c | *dusB* | 2.624 | tRNA dihydrouridine synthase DusB |
| SMU.2036 |  | 2.639 | peptidase |
| SMU.2037 | *treC* | 2.214 | alpha,alpha-phosphotrehalase |
| SMU.2038 | *treB* | 2.449 | PTS system trehalose-specific EIIBC component |
| SMU.2046c |  | 2.184 | endonuclease/exonuclease/phosphatase  family protein |
| SMU.2047 | *malT* | 2.270 | PTS transporter subunit IIBC |
| SMU.2117 |  | 2.489 | ABC transporter permease |
| SMU.2118 |  | 2.555 | osmoprotectant ABC transporter substrate-  binding protein |
| SMU.2119 |  | 2.959 | ABC transporter permease |
| SMU.RS08485 |  | 2.256 | hypothetical protein |
| SMU.RS10120 |  | 2.320 | SPJ_0845 family protein |

**Table S2.** **Down-regulated genes in DE dataset.**

| Locus Tag | Gene name | Fold change | Description |  |
| --- | --- | --- | --- | --- |
| SMU.01 | *dnaA* | 0.339 | chromosomal replication initiator protein DnaA |  |
| SMU.05 |  | 0.417 | DUF951 domain-containing protein |  |
| SMU.07 | *pth* | 0.195 | aminoacyl-tRNA hydrolase |  |
| SMU.13 | *tilS* | 0.145 | tRNA lysidine synthetase TilS |  |
| SMU.14 | *hpt* | 0.188 | hypoxanthine phosphoribosyltransferase |  |
| SMU.20 | *mreC* | 0.238 | rod shape-determining protein MreC |  |
| SMU.21 | *mreD* | 0.304 | rod shape-determining protein MreD |  |
| SMU.40 |  | 0.303 | type II toxin-antitoxin system mRNA interferase  toxin, Rel/StbE family |  |
| SMU.42 |  | 0.455 | Abi family protein |  |
| SMU.63c |  | 0.495 | carbohydrate-binding domain-containing protein |  |
| SMU.80 | *hrcA* | 0.139 | heat-inducible transcriptional repressor HrcA |  |
| SMU.81 | *grpE* | 0.137 | nucleotide exchange factor GrpE |  |
| SMU.82 | *dnaK* | 0.198 | molecular chaperone DnaK |  |
| SMU.83 | *dnaJ* | 0.346 | molecular chaperone DnaJ |  |
| SMU.96 | *rpoE* | 0.383 | DNA-directed RNA polymerase subunit delta |  |
| SMU.110 |  | 0.208 | Rgg/GadR/MutR family transcriptional regulator |  |
| SMU.113 | *pfkB* | 0.421 | 1-phosphofructokinase |  |
| SMU.116 | *lacD* | 0.476 | tagatose-bisphosphate aldolase |  |
| SMU.120 | *rpmB* | 0.294 | 50S ribosomal protein L28 |  |
| SMU.121 |  | 0.330 | MATE family efflux transporter |  |
| SMU.152 |  | 0.349 | hypothetical protein |  |
| SMU.172 |  | 0.372 | AbrB/MazE/SpoVT family DNA-binding domain-  Containing protein |  |
| SMU.174c |  | 0.280 | nucleotidyltransferase domain-containing protein |  |
| SMU.222c |  | 0.175 | hypothetical protein |  |
| SMU.223c |  | 0.263 | DUF771 domain-containing protein |  |
| SMU.224c |  | 0.262 | DUF771 domain-containing protein |  |
| SMU.236c |  | 0.352 | TetR/AcrR family transcriptional regulator |  |
| SMU.241c |  | 0.484 | amino acid ABC transporter ATP-binding protein |  |
| SMU.279 |  | 0.457 | hypothetical protein |  |
| SMU.281 |  | 0.470 | hypothetical protein |  |
| SMU.287 |  | 0.279 | HlyD family efflux transporter periplasmic  adaptor subunit |  |
| SMU.333 |  | 0.268 | hypothetical protein |  |
| SMU.334 |  | 0.404 | argininosuccinate synthase |  |
| SMU.338 |  | 0.362 | protein jag |  |
| SMU.354 |  | 0.315 | DNA recombination protein RmuC |  |
| SMU.372 |  | 0.196 | hypothetical protein |  |
| SMU.373 |  | 0.252 | methyltransferase domain-containing protein |  |
| SMU.374 |  | 0.376 | SDR family oxidoreductase |  |
| SMU.375 |  | 0.390 | hypothetical protein |  |
| SMU.376 |  | 0.313 | aminotransferase class III-fold pyridoxal  phosphate-dependent enzyme |  |
| SMU.393 |  | 0.349 | DUF536 domain-containing protein |  |
| SMU.394c |  | 0.449 | YbaB/EbfC family nucleoid-associated protein |  |
| SMU.405c |  | 0.492 | helix-turn-helix domain-containing protein |  |
| SMU.447 |  | 0.160 | DUF896 family protein |  |
| SMU.448 |  | 0.168 | PepSY domain-containing protein |  |
| SMU.459 |  | 0.308 | transporter substrate-binding domain-containing  protein |  |
| SMU.470 |  | 0.481 | DUF1273 domain-containing protein |  |
| SMU.490 |  | 0.363 | glycyl-radical enzyme activating protein |  |
| SMU.491 |  | 0.314 | DeoR/GlpR family DNA-binding transcription  regulator |  |
| SMU.499 |  | 0.330 | ComF family protein |  |
| SMU.501 |  | 0.369 | hypothetical protein |  |
| SMU.503c |  | 0.280 | hypothetical protein |  |
| SMU.505 |  | 0.238 | site-specific DNA-methyltransferase |  |
| SMU.506 |  | 0.228 | type II restriction endonuclease |  |
| SMU.514 |  | 0.330 | TetR/AcrR family transcriptional regulator |  |
| SMU.539c |  | 0.428 | A24 family peptidase |  |
| SMU.550 |  | 0.311 | FtsQ-type POTRA domain-containing protein |  |
| SMU.556 |  | 0.220 | RNA-binding protein |  |
| SMU.557 |  | 0.420 | DivIVA domain-containing protein |  |
| SMU.560c |  | 0.173 | DUF1827 family protein |  |
| SMU.561c |  | 0.420 | NUDIX hydrolase |  |
| SMU.563 | *argF* | 0.430 | ornithine carbamoyltransferase |  |
| SMU.587 |  | 0.437 | SGNH/GDSL hydrolase family protein |  |
| SMU.588 |  | 0.350 | YpmS family protein |  |
| SMU.602 |  | 0.496 | bile acid:sodium symporter family protein |  |
| SMU.609 |  | 0.226 | SH3 domain-containing protein |  |
| SMU.682 |  | 0.165 | dynamin family protein |  |
| SMU.688 |  | 0.317 | YkgJ family cysteine cluster protein |  |
| SMU.690 |  | 0.340 | DUF6287 domain-containing protein |  |
| SMU.697 | *infC* | 0.277 | translation initiation factor IF-3 |  |
| SMU.700c |  | 0.304 | histidine phosphatase family protein |  |
| SMU.701c |  | 0.368 | YccF domain-containing protein |  |
| SMU.717 |  | 0.361 | aminoacyltransferase |  |
| SMU.718c | *yidA* | 0.371 | sugar-phosphatase |  |
| SMU.720 |  | 0.426 | sulfite exporter TauE/SafE family protein |  |
| SMU.721 |  | 0.234 | DUF1934 domain-containing protein |  |
| SMU.730 |  | 0.235 | AbrB/MazE/SpoVT family DNA-binding domain-  Containing protein |  |
| SMU.731 |  | 0.276 | ABC transporter ATP-binding protein |  |
| SMU.753 |  | 0.267 | PspC domain-containing protein |  |
| SMU.758c |  | 0.341 | DUF3270 domain-containing protein |  |
| SMU.768c |  | 0.194 | hypothetical protein |  |
| SMU.804 |  | 0.230 | FRG domain-containing protein |  |
| SMU.813 |  | 0.365 | helix-turn-helix domain-containing protein |  |
| SMU.815 |  | 0.197 | transporter substrate-binding domain-containing  protein |  |
| SMU.817 |  | 0.296 | transporter substrate-binding domain-containing  protein |  |
| SMU.818 | *rpsU* | 0.478 | 30S ribosomal protein S21 |  |
| SMU.820 |  | 0.454 | hypothetical protein |  |
| SMU.821 | *dnaG* | 0.485 | DNA primase |  |
| SMU.840c |  | 0.332 | hypothetical protein |  |
| SMU.846 | *rplU* | 0.398 | 50S ribosomal protein L21 |  |
| SMU.862 |  | 0.270 | efflux RND transporter periplasmic adaptor subunit |  |
| SMU.863 |  | 0.314 | ABC transporter ATP-binding protein |  |
| SMU.865 | *rpsP* | 0.484 | 30S ribosomal protein S16 |  |
| SMU.870 |  | 0.409 | DeoR family transcriptional regulator |  |
| SMU.890 |  | 0.453 | phosphatase PAP2 family protein |  |
| SMU.891 |  | 0.438 | type I restriction-modification system subunit M |  |
| SMU.893 |  | 0.164 | AAA family ATPase |  |
| SMU.895 |  | 0.317 | type II toxin-antitoxin system RelB/DinJ family  antitoxin |  |
| SMU.897 |  | 0.323 | type I restriction endonuclease subunit R |  |
| SMU.930c |  | 0.335 | LysR family transcriptional regulator |  |
| SMU.932 |  | 0.272 | uroporphyrinogen decarboxylase family protein |  |
| SMU.933 |  | 0.336 | amino acid ABC transporter substrate-binding  protein |  |
| SMU.934 |  | 0.374 | amino acid ABC transporter permease |  |
| SMU.935 |  | 0.371 | amino acid ABC transporter permease |  |
| SMU.936 |  | 0.401 | amino acid ABC transporter ATP-binding protein |  |
| SMU.941c |  | 0.305 | DUF1836 domain-containing protein |  |
| SMU.947 |  | 0.169 | dihydrofolate reductase |  |
| SMU.948 |  | 0.266 | hypothetical protein |  |
| SMU.949 | *clpX* | 0.359 | ATP-dependent Clp protease ATP-binding subunit  ClpX |  |
| SMU.950 | *yihA* | 0.405 | ribosome biogenesis GTP-binding protein  YihA/YsxC |  |
| SMU.956 |  | 0.448 | ATP-dependent Clp protease ATP-binding subunit |  |
| SMU.983 |  | 0.465 | AraC family transcriptional regulator |  |
| SMU.984 |  | 0.225 | CHAP domain-containing protein |  |
| SMU.987 |  | 0.373 | Ig-like domain-containing protein |  |
| SMU.988 | *cls* | 0.446 | cardiolipin synthase |  |
| SMU.997 |  | 0.293 | ATP-binding cassette domain-containing protein |  |
| SMU.998 |  | 0.241 | siderophore ABC transporter substrate-binding  protein |  |
| SMU.999 |  | 0.463 | hypothetical protein |  |
| SMU.1002 | *topA* | 0.423 | type I DNA topoisomerase |  |
| SMU.1027 |  | 0.317 | TetR/AcrR family transcriptional regulator |  |
| SMU.1028 |  | 0.244 | alpha/beta hydrolase |  |
| SMU.1034c | *xerS* | 0.189 | tyrosine recombinase XerS |  |
| SMU.1037c |  | 0.298 | HAMP domain-containing histidine kinase |  |
| SMU.1038c |  | 0.234 | response regulator transcription factor |  |
| SMU.1052 |  | 0.208 | DUF1831 domain-containing protein |  |
| SMU.1061 |  | 0.411 | putative DNA-binding protein |  |
| SMU.1069c |  | 0.346 | DUF3021 family protein |  |
| SMU.1070c |  | 0.241 | LytTR family transcriptional regulator DNA-  binding domain-containing protein |  |
| SMU.1080c |  | 0.349 | lysozyme family protein |  |
| SMU.1081c |  | 0.278 | nucleoid-associated protein |  |
| SMU.1097c |  | 0.369 | MarR family transcriptional regulator |  |
| SMU.1107c |  | 0.326 | SGNH/GDSL hydrolase family protein |  |
| SMU.1111c |  | 0.403 | DUF1002 domain-containing protein |  |
| SMU.1126 | *coaA* | 0.356 | type I pantothenate kinase |  |
| SMU.1127 | *rpsT* | 0.250 | 30S ribosomal protein S20 |  |
| SMU.1128 | *ciaH* | 0.296 | three-component system sensor histidine kinase  CiaH |  |
| SMU.1129 | *ciaR* | 0.284 | three-component system response regulator CiaR |  |
| SMU.1131c | *ciaX* | 0.389 | three-component system regulator CiaX |  |
| SMU.1133 | *phoU* | 0.361 | phosphate signaling complex protein PhoU |  |
| SMU.1135 | *pstB* | 0.443 | phosphate ABC transporter ATP-binding protein  PstB |  |
| SMU.1157c |  | 0.279 | SIR2 family protein |  |
| SMU.1177c |  | 0.302 | amino acid ABC transporter substrate-binding  protein |  |
| SMU.1178c |  | 0.262 | amino acid ABC transporter ATP-binding protein |  |
| SMU.1179c |  | 0.427 | amino acid ABC transporter permease |  |
| SMU.1188 | *lepB* | 0.380 | signal peptidase I |  |
| SMU.1205c |  | 0.205 | hypothetical protein |  |
| SMU.1206c |  | 0.238 | GTP pyrophosphokinase |  |
| SMU.1209c |  | 0.334 | hypothetical protein |  |
| SMU.1237c |  | 0.418 | nuclear transport factor 2 family protein |  |
| SMU.1249c |  | 0.290 | DUF4352 domain-containing protein |  |
| SMU.1259 |  | 0.397 | Eco57I restriction-modification methylase  domain-containing protein |  |
| SMU.1261 | *hisE* | 0.446 | phosphoribosyl-ATP diphosphatase |  |
| SMU.1262c |  | 0.237 | hypothetical protein |  |
| SMU.1263 | *hisI* | 0.482 | phosphoribosyl-AMP cyclohydrolase |  |
| SMU.1302 |  | 0.488 | ZinT/AdcA family metal-binding protein |  |
| SMU.1315c |  | 0.383 | AAA family ATPase |  |
| SMU.1316c |  | 0.350 | hypothetical protein |  |
| SMU.1317c |  | 0.254 | hypothetical protein |  |
| SMU.1334 | *mubP* | 0.257 | mutanobactin A biosynthesis phosphopantetheinyl  transferase MubP |  |
| SMU.1335c | *mubJ* | 0.498 | mutanobactin A biosynthesis reductase MubJ |  |
| SMU.1339 | *mubD* | 0.224 | mutanobactin A non-ribosomal peptide synthetase  MubD |  |
| SMU.1340 | *mubC* | 0.225 | mutanobactin A non-ribosomal peptide synthetase  MubC |  |
| SMU.1341c | *mubB* | 0.272 | mutanobactin A non-ribosomal peptide synthetase  MubB |  |
| SMU.1342 | *mubA* | 0.249 | mutanobactin A non-ribosomal peptide synthetase  MubA |  |
| SMU.1349 | *mubR* | 0.206 | mutanobactin A biosynthesis transcriptional  regulator MubR |  |
| SMU.1391c |  | 0.411 | DUF6287 domain-containing protein |  |
| SMU.1392c |  | 0.245 | GNAT family N-acetyltransferase |  |
| SMU.1399 | *irvR* | 0.420 | LexA family transcriptional regulator IrvR |  |
| SMU.1402c | *csn2* | 0.375 | type II-A CRISPR-associated protein Csn2 |  |
| SMU.1405c | *cas9* | 0.267 | type II CRISPR RNA-guided endonuclease Cas9 |  |
| SMU.1406c |  | 0.479 | NADPH-dependent oxidoreductase |  |
| SMU.1442c |  | 0.138 | YehR family protein |  |
| SMU.1447c |  | 0.438 | ABC transporter substrate-binding protein |  |
| SMU.1470c |  | 0.247 | GyrI-like domain-containing protein |  |
| SMU.1495 | *lacB* | 0.296 | galactose-6-phosphate isomerase subunit LacB |  |
| SMU.1498 | *lacR* | 0.456 | transcriptional regulator LacR |  |
| SMU.1504c |  | 0.485 | hypothetical protein |  |
| SMU.1509 |  | 0.240 | Rgg/GadR/MutR family transcriptional regulator |  |
| SMU.1519 |  | 0.308 | amino acid ABC transporter ATP-binding protein |  |
| SMU.1520 |  | 0.470 | transporter substrate-binding domain-containing  protein |  |
| SMU.1595 |  | 0.361 | carbonic anhydrase family protein |  |
| SMU.1600 |  | 0.200 | PTS cellobiose transporter subunit IIB |  |
| SMU.1604c |  | 0.366 | PadR family transcriptional regulator |  |
| SMU.1610 | *rpmG* | 0.267 | 50S ribosomal protein L33 |  |
| SMU.1610 | *rpmG* | 0.472 | 50S ribosomal protein L33 |  |
| SMU.1624 | *frr* | 0.499 | ribosome recycling factor |  |
| SMU.1631 |  | 0.360 | peptidylprolyl isomerase |  |
| SMU.1642c |  | 0.345 | DUF421 domain-containing protein |  |
| SMU.1644c |  | 0.320 | hypothetical protein |  |
| SMU.1671c |  | 0.196 | YlbF family regulator |  |
| SMU.1672 | *clpP* | 0.491 | AP-dependent Clp protease proteolytic subunit  lpP |  |
| SMU.1704 |  | 0.317 | PadR family transcriptional regulator |  |
| SMU.1705 |  | 0.352 | DUF1700 domain-containing protein |  |
| SMU.1706 |  | 0.365 | DUF4097 family beta strand repeat-containing  protein |  |
| SMU.1707c |  | 0.360 | tRNA (cytidine(34)-2'-O)-methyltransferase |  |
| SMU.1712c | *scpB* | 0.445 | SMC-Scp complex subunit ScpB |  |
| SMU.1713c |  | 0.334 | segregation/condensation protein A |  |
| SMU.1714c | *xerD* | 0.420 | site-specific tyrosine recombinase XerD |  |
| SMU.1728 | *greA* | 0.332 | transcription elongation factor GreA |  |
| SMU.1730c |  | 0.402 | GNAT family N-acetyltransferase |  |
| SMU.1732c |  | 0.207 | hypothetical protein |  |
| SMU.1743 |  | 0.377 | acyl carrier protein |  |
| SMU.1760c | *cas7c* | 0.456 | type I-C CRISPR-associated protein Cas7/Csd2 |  |
| SMU.1764c |  | 0.120 | CRISPR-associated helicase/endonuclease Cas3 |  |
| SMU.1765c |  | 0.348 | WYL domain-containing protein |  |
| SMU.1773c |  | 0.461 | SMEK domain-containing protein |  |
| SMU.1781 |  | 0.404 | DUF402 domain-containing protein |  |
| SMU.1803c |  | 0.225 | DUF4230 domain-containing protein |  |
| SMU.1855 | *hdrM* | 0.408 | hdrR negative regulator HdrM |  |
| SMU.1861c |  | 0.324 | hypothetical protein |  |
| SMU.1872c |  | 0.443 | hypothetical protein |  |
| SMU.1904c |  | 0.184 | thioredoxin family protein |  |
| SMU.1922 |  | 0.246 | DnaD domain protein |  |
| SMU.1923c | *nrdR* | 0.363 | transcriptional regulator NrdR |  |
| SMU.1925c |  | 0.398 | YceD family protein |  |
| SMU.1926 |  | 0.272 | TetR/AcrR family transcriptional regulator |  |
| SMU.1931 | *rsmG* | 0.489 | 16S rRNA (guanine(527)-N(7))-methyltransferase  RsmG |  |
| SMU.1941 |  | 0.222 | MetQ/NlpA family ABC transporter substrate-  binding protein |  |
| SMU.1942c |  | 0.178 | amino acid ABC transporter substrate-binding  protein |  |
| SMU.1963c |  | 0.348 | extracellular solute-binding protein |  |
| SMU.1966c |  | 0.348 | sugar ABC transporter substrate-binding protein |  |
| SMU.1969c |  | 0.230 | MarR family transcriptional regulator |  |
| SMU.1976c |  | 0.368 | hypothetical protein |  |
| SMU.1977c |  | 0.283 | helix-turn-helix transcriptional regulator |  |
| SMU.1981c | *comGF* | 0.354 | competence type IV pilus minor pilin ComGF |  |
| SMU.1982c | *comGE* | 0.362 | competence type IV pilus minor pilin ComGE |  |
| SMU.1983 | *comGD* | 0.329 | competence type IV pilus minor pilin ComGD |  |
| SMU.1984 | *comGC* | 0.446 | competence type IV pilus major pilin ComGC |  |
| SMU.1985 | *comGB* | 0.297 | competence type IV pilus assembly protein  ComGB |  |
| SMU.1987 | *comGA* | 0.298 | competence type IV pilus ATPase ComGA |  |
| SMU.1992 | *tyrS* | 0.488 | tyrosine--tRNA ligase |  |
| SMU.1999c |  | 0.447 | glutamate-cysteine ligase family protein |  |
| SMU.2000 | *rplQ* | 0.330 | 50S ribosomal protein L17 |  |
| SMU.2001 |  | 0.350 | DNA-directed RNA polymerase subunit alpha |  |
| SMU.2002 | *rpsK* | 0.328 | 30S ribosomal protein S11 |  |
| SMU.2003 | *rpsM* | 0.337 | 30S ribosomal protein S13 |  |
| SMU.2003a | *rpmJ* | 0.293 | 50S ribosomal protein L36 |  |
| SMU.2004 | *infA* | 0.298 | translation initiation factor IF-1 |  |
| SMU.2033c |  | 0.274 | hypothetical protein |  |
| SMU.2060 |  | 0.437 | LysR family transcriptional regulator |  |
| SMU.2061 |  | 0.457 | DUF4947 domain-containing protein |  |
| SMU.2077c |  | 0.416 | DUF1292 domain-containing protein |  |
| SMU.2080 | *brsR* | 0.308 | bacteriocin genes transcriptional regulator BrsR |  |
| SMU.2081 | *brsM* | 0.155 | bacteriocin genes regulator BrsM |  |
| SMU.2084c | *spxA2* | 0.098 | transcriptional regulator Spx |  |
| SMU.2085 | *recA* | 0.460 | recombinase RecA |  |
| SMU.2087 |  | 0.308 | DNA-3-methyladenine glycosylase I |  |
| SMU.2088 | *ruvA* | 0.271 | Holliday junction branch migration protein RuvA |  |
| SMU.2105 | *rpmF* | 0.202 | 50S ribosomal protein L32 |  |
| SMU.2106c |  | 0.418 | transcriptional regulator |  |
| SMU.2107c |  | 0.461 | hypothetical protein |  |
| SMU.2108c |  | 0.359 | DUF5937 family protein |  |
| SMU.2113c |  | 0.252 | histidine phosphatase family protein |  |
| SMU.2114c |  | 0.191 | MerR family transcriptional regulator |  |
| SMU.2134 |  | 0.490 | TetR/AcrR family transcriptional regulator |  |
| SMU.2137c | *sprV* | 0.275 | transcriptional regulator SprV |  |
| SMU.2139c | *rplI* | 0.318 | 50S ribosomal protein L9 |  |
| SMU.2140c |  | 0.408 | DHH family phosphoesterase |  |
| SMU.2154c |  | 0.265 | insulinase family protein |  |
| SMU.2158c | *trpS* | 0.403 | tryptophan--tRNA ligase |  |
| SMU.2162c | *rlmH* | 0.247 | 23S rRNA (pseudouridine(1915)-N(3))-  methyltransferase RlmH |  |
| SMU.2164 |  | 0.290 | trypsin-like peptidase domain-containing protein |  |
| SMU.RS00765 |  | 0.135 | winged helix-turn-helix transcriptional regulator | |
| SMU.RS02435 |  | 0.492 | hypothetical protein | |
| SMU.RS02465 |  | 0.240 | hypothetical protein | |
| SMU.RS04735 |  | 0.270 | cysteine-rich KTR domain-containing protein | |
| SMU.RS06070 |  | 0.250 | hypothetical protein | |
| SMU.RS06380 |  | 0.364 | type II toxin-antitoxin system HicA family toxin | |
| SMU.RS09515 |  | 0.170 | hypothetical protein | |
| SMU.RS10145 |  | 0.442 | ClbS/DfsB family four-helix bundle protein | |

**Table S3.** **List of strains, plasmids and primers used in this study.**

| Strains, plasmids or primers | | | Genotype or sequence | | Reference | | | | | | |
| --- | --- | --- | --- | --- | --- | --- | --- | --- | --- | --- | --- |
| *Streptococcus mutans* | | | | |  | | | | | | |
| UA159 | | Wild Type | | Laboratory Stock | | | | | | | |
| ∆SMU.1147 | | ∆SMU.1147::npKm | | Laboratory Stock (24) | | | | | | | |
| SDK001 | | pIB184::SMU.1147 in ∆SMU.1147 | | This work | | | | | | | |
| Plasmids | |  | |  | | | | | | | |
| pIB184 | | *E. coli-Streptococcus* shuttle vector, Ery^r^ | | Laboratory Stock | | | | | | | |
| pDL278 | | *E. coli-Streptococcus* shuttle vector, Sp^r^ | | Laboratory Stock | | | | | | | |
| pDK001 | | SMU.1147 cloned into pIB184 | | This work | | | | | | | |
| Primers | | |  | | |  | | | | |  |
| SMU.1147c_FP-*BamHI* | | 5’-TTTTTTGGATCCAGCCAGCTCATGATTGA-3’ | | | | | | | | |  |
| SMU.1147c_RP-*EcoRI* | | 5’-TTTTTTGAATTCACGCCTGTTAGGTTGTTA -3’ | | | | | | | | |  |
| RT_ComC_FP | | 5’-GAGATTATCATTGGCGGAA-3’ | | | |  | | | | |  |
| RT_ComC_RP | | 5’-CCCAAAGCTTGTGTAAAAC-3’ | | | |  | | | | |  |
| RT_ComD_FP | | 5’-CTCTTTCTCAGTGTGTT-3’ | | | |  | | | | |  |
| RT_ComD_RP | | 5’-CATTGGAATCAAGCGTT-3’ | | | |  | | | | |  |
| RT_ComE_FP | | 5’-CCCTTTTGCTGAGATTC-3’ | | | |  | | | | |  |
| RT_ComE_RP | | 5’-TAACAATGTCAGTCATACT-3’ | | | |  | | | | |  |
| RT_ComX_FP | | 5’-CGTCAGCAAGAAAGTCAGAAAC-3’ | | | |  | | | | |  |
| RT_ComX_RP | | 5’-ATACCGCCACTTGACAAACAG-3’ | | | |  | | | | |  |
| RT_ComR_FP | | 5’-TATTACGAAGGCCAACCTAT-3’ | | | |  | | | | |  |
| RT_ComR_RP | | 5’-TTCTTCTTCAGGCAAATGAT-3’ | | | |  | | | | |  |
| RT_ScnK_FP | | 5’-TCAGGCTACACCAGTCAATTG-3’ | | | |  | | | | |  |
| RT_ScnK_RP | | 5’-GACGCTTTTGATTATCCAACAGG-3’ | | | |  | | | | |  |
| RT_ScnR_FP | | 5’-GTTTTGAGGTACTAAGAGAAATTCGG-3’ | | | | | | |  | |  |
| RT_ScnR_RP | | 5’-GGACGAAAAGGTTTGACGATG-3’ | | | |  | | | | |  |
| RT_CiaH_FP | | 5’-ATAAAGCCAGAACACGACAAAAG-3’ | | | | |  | | | |  |
| RT_CiaH_RP | | 5’-CAGGCGAGCTTCAAACATTAC-3’ | | | |  | | | | |  |
| RT_CiaR_FP | | 5’-TTGATCGTATCTGGGGCTTTG-3’ | | | |  | | | | |  |
| RT_CiaR_RP | | 5’-TGCGTAATGTCTGTAAATTCTTTCC-3’ | | | | |  | | | |  |
| RT_CiaX_FP | | 5’-CAAAATTAACTCAGACGAAGCCTAC-3’ | | | | | |  | | |  |
| RT_CiaX_RP | | 5’-GGAATCACTATCCGATTGCGA-3’ | | | | |  | | | |  |
| RT_HtrA_FP | | 5’-CAATTATGACAGCGGTTCTCAAG-3’ | | | | |  | | | |  |
| RT_HtrA_RP | | 5’-GTAACTAAATAAGCACTATCGCCATC-3’ | | | | | |  | | |  |
| RT_16s rRNA_FP | | 5’-CACACCGCCCGTCACACC-3’ | | | | | |  | | |  |
| RT_16s rRNA_RP | | 5’-CAGCCGCACCTTCCGATACG-3’ | | | | | |  | | |  |
| RT_SMU.675_FP | | 5’-AGGTAATCAGATGTTCCGCAC-3’ | | | | | |  | | |  |
| RT_SMU.675_RP | | 5’-TGGGAACATGATACGCAACTG-3’ | | | | | |  | | |  |
| RT_SMU.129_FP | | 5’-CGTGATCTGGGAGTTAACCTG-3’ | | | | | |  | | |  |
| RT_SMU.129_RP | | 5’-CAACATCTTCCTTGTGAACACG-3’ | | | | | |  | | |  |
| RT_SMU.1421_FP | | 5’-CTGAAAAGCATGAAGACGGAC-3’ | | | | | |  | | |  |
| RT_SMU.1421_RP | | 5’-GCAGGATTAAAGGCTTCAACG-3’ | | | | | |  | | |  |
| RT-SMU.1147-FP | | 5’-CGTTTAGTGTCCTGTCATTCCCTTC-3’ | | | | | | | | |  |
| RT-SMU.1147-RP | | 5‘-TCTGATCCAAGGGCTTCAAAGCAA-3’ | | | | | | | | |  |

**Table S4. RNA-seq read counts and mapping efficiency.**

| Sample name | Processed read | Mapped read | Mapping rate |
| --- | --- | --- | --- |
| WT-1 | 48376838 | 34948237 | 72.24% |
| WT-2 | 47458732 | 47395474 | 99.87% |
| WT-3 | 48229286 | 34878985 | 72.32% |
| ΔSMU.1147-1 | 47557104 | 46036390 | 96.80% |
| ΔSMU.1147-2 | 47885826 | 47778490 | 99.78% |
| ΔSMU.1147-3 | 48008200 | 47893364 | 99.76% |
